# Identification and Expression Profiling of miRNAome in Goat *longissimus dorsi* Muscle from Prenatal Stages to a Neonatal Stage

**DOI:** 10.1371/journal.pone.0165764

**Published:** 2016-10-31

**Authors:** Jiazhong Guo, Wei Zhao, Siyuan Zhan, Li Li, Tao Zhong, Linjie Wang, Yao Dong, Hongping Zhang

**Affiliations:** College of Animal Science and Technology, Sichuan Agricultural University, Chengdu, Postcode 611130, People’s Republic of China; Kunming University of Science and Technology, CHINA

## Abstract

Skeletal muscle development is a complex biological process regulated by numerous genes and non-coding RNAs, such as microRNAs (miRNAs). In the current study, we made use of the deep sequencing data from Jianzhou Da’er goat *longissimus dorsi* sampled on days 45, 60, and 105 of gestation, as well as day three after birth to identify miRNAs that regulate goat skeletal myogenesis, and examine their temporal expression profiles. A total of 410 known goat miRNAs, 752 miRNA homologs and 88 novel miRNAs were identified across four stages. Besides three myomiRs, the abundance of 17 miRNAs, including chi-miR-424, chi-miR-542-3p and chi-miR-136-5p was more than 10,000 reads per million mapped reads (RPM), on average. Furthermore, 50 miRNAs with more than 100 RPM clustered at the imprinted *DLK1–DIO3* locus on chromosome 21 and showed similar expression patterns, indicating that these miRNAs played important roles in skeletal myogenesis of goats. Based on pairwise comparisons, 221 differentially expressed (DE), known miRNAs were identified across four stages. GO and KEGG analyses of the genes targeted by the DE miRNAs revealed the significantly enriched processes and pathways to be consistent with temporal changes of skeletal muscle development across all sampled stages. However, follow-up experimental studies were required to explore functions of these miRNAs and targets underlying skeletal myogenesis.

## Introduction

MicroRNAs (miRNAs) are defined as a class of small, non-coding RNAs, typically with a length of ~ 22 nucleotides, that are synthesized endogenously in higher organisms. As a post-transcriptional regulatory element, miRNA action is mainly accomplished by interfering with the translation process of the protein-coding genes via base pairing of the seed sequence with the 3´UTR and CDS regions of their target genes [1]. Studies over the past years have revealed, that miRNAs are ubiquitously expressed in animal tissues and play key roles in a variety of biological processes, e.g. myogenic differentiation [2, 3], neurogenesis [4], vascular remodeling [5], lipid metabolism [6] and glucose homeostasis [7, 8]. To date, the number of newly identified miRNAs is still growing. Thus, it is assumed that the biological functions of these small non-coding RNAs are likely beyond our current understanding.

Domesticated animals, such as pig, cattle, and goat, primarily serve as a major source of meat, which is mainly composed of muscle and fat. It has been widely recognized that the prenatal stage is critical for skeletal muscle development, as the formation of muscle fibers generally occurs prior to birth [9]. From a genetical point of view, skeletal muscle development is considered a complicated biological process regulated by numerous genes, including *PAX3*, *PAX7*, *MyoD* and *Myf-5* [10]. Furthermore, increasing attention has been focused on the functional importance of miRNAs in the regulation of skeletal myogenesis [11]. In studies of livestock [12–17], transcriptome and miRNAome have provided new insights into the mechanisms of skeletal muscle development, particularly for pig. For instance, Zhao et al. described the differential expression of genes, like *GSK3B*, *IKBKB*, and *ACVR1*, which are associated with myogenesis in both obese and lean pig breeds [13]. In addition to the myomiRs (miR-1, miR-133 and miR-206), other miRNAs, such as miR-542-3p and let-7a, are highly expressed in skeletal muscle throughout the prenatal and neonatal periods, suggesting that other miRNAs also play important roles in regulating pig myogenesis [14]. The multifaceted roles of miRNAs in muscle growth are also true for poultry, as evidenced by a comparative analysis revealing interaction of several miRNAs with the *ACVR2B* gene, resulting in distinct muscle development of broiler and layer chickens [16]. Moreover, the regulation of the *myostatin* gene may be connected with the synthesis of miR-1 and miR-206, which directly affects muscularity in Belgian Texel sheep [15]. For myogenesis, these studies not only represent the molecular basis necessary to understand skeletal muscle development and morphogenesis, but also broaden our understanding of the metabolic activity that influences meat production in the livestock industry.

With the successful applications of high-throughput sequencing technologies, a large number of miRNAs regulating the development of the mammary glands, skin growth, and the hair follicle formation cycle have been identified in goats [18–20]. However, the number of goat miRNA sequences in miRBase is much lower compared to the data deposition of miRNAs for pig and cattle. Wang et al. [21] recently examined the genome-wide expression profiles of miRNAs and their interactions with mRNAs in the Xuhuai goat. They used *Longissimus thoracis* samples from a 90-day fetus and a six month-old goat, and their results suggested miRNAs to play important roles during goat skeletal muscle development. However, more work is required to decipher the comprehensive muscle-related miRNAome in goat by documenting multiple developmental stages, particularly during prenatal stages.

The Jianzhou Da’er goat is a Chinese goat breed, recently developed in Sichuan for meat production. This breed is well-known in southwest China for its fast growth rate and high meat percentage. In this study, we used deep sequencing data from the Jianzhou Da’er goat *longissimus dorsi* samples on days 45, 60, and 105 of gestation, as well as day three after birth. We systematically identified miRNAs involved in goat skeletal muscle development, and investigated their temporal expression profiles from an early prenatal stage to a neonatal stage.

## Materials and Methods

### Animal ethics statement

All experimental procedures involving animals were conducted in accordance with the Regulations for the Administration of Affairs Concerning Experimental Animals (Ministry of Science and Technology, China) and were approved by the Institutional Animal Care and Use Committee of the College of Animal Science and Technology, Sichuan Agricultural University, Sichuan, China.

Non-steroidal anti-inflammatory drug (FLUNDAN MEGLUMINE INJECTION, Hebei Yuanzheng Pharmaceutical Co., Ltd, China) was used to prevent wound inflammation and pain for three days, after the caesarean section. The sampled ewes were also monitored their health status, which included body temperature, pulse measurement for heart rate and chewing activity. There was no serious adverse events occurred for these ewes. All the sampled individuals (embryos, fetus, and newborns) were euthanized by electrical stunning followed by exsanguination, if necessary.

### Animal and sample preparation

The sampled pregnant Jianzhou Da’er ewes were raised at the same free-stall housing and fed a standard diet on the Jianyang Dageda farm (Sichuan, China). These Ewes were fed a standard diet (forage to concentrate ratio, 65:35) twice per day at 06:30–08:30 and 16:00–18:00, and water *ad libitum*. Three sampled pregnant ewes at each development stage were then subjected to caesarean section for collection of the muscle samples from embryos or fetuses. Ultimately, the *longissimus dorsi* muscle samples were collected at four developmental stages of skeletal muscle, including three prenatal stages (days 45, 60, and 105 of gestation of the ewes, denoted as E45, E60 and E105, respectively) and one neonatal stage (the three-day-old newborn, denoted as B3) from the Jianzhou Da’er goats. Considering the variability of biological samples, two samples per stage (E45-1 and E45-2 for E45, E60-1 and E60-2 for E60, E105-1 and E105-2 for E105, and B3-1 and B3-2 for B3) were used for deep sequencing analysis. The eight sampled goat individuals at the four time points were from different estrus-synchronized ewes. Sex determination via PCR amplification of the SRY gene indicated that all sampled animals were female.

### Small RNA library construction and sequencing

Total RNA was extracted from each of the eight muscle samples using the miRNeasy kit (QIAGEN, Germany) following the manufacturer’s protocol. The Agilent 2100 Bioanalyzer (Agilent Technologies, USA) was used to quantify the total RNA integrity after initial quality measurement with a NanoDrop 2000 (NanoDrop Technologies, USA). The RNA samples, which had RIN values ranging from 7.3 to 8.4, were then used for small RNA enrichment. Small RNA fragments were ligated to 5´ and 3´ adaptors using T4 RNA ligase, followed by reverse transcription PCR. Subsequently, only the products with lengths between 40–160 nt were isolated from the agarose gel. Multiplexed libraries were finally subjected to 50 bp single-end sequencing on an Illumina HiSeq 2500 sequencer.

### Computational analysis of small RNA sequences

Clean reads were obtained after remove of low quality reads (those that contain more than one unknown (N) base or more than one base showing a Phred score of less than 20) and removing reads with 5' linker sequence, reads without 3' linker sequence, reads without the insert tag, reads with poly (A), and reads shorter than 16 nt. Subsequently, we summarized the length distribution of these clean reads to examine the quality of our sequence data. As some sRNA sequences could possibly have been mapped to more than one type during the annotation of the clean reads, we employed the following priority criteria to classify each sRNA sequence into only one category: rRNA, etc. (Genbank > Rfam) > known miRNA > repeat > exon > novel miRNA > intron.

The clean reads were compared to the Genbank for the first time and Rfam databases to filter out the non-coding RNAs (rRNAs, tRNAs, snRNAs, and snoRNA) deposited in the NCBI GenBank and the Rfam 10.1 databases, to remove various types of non-coding RNAs, mainly including rRNAs and tRNAs. To identify known goat miRNAs, the remaining clean reads were aligned to the goat miRNA precursors present in miRBase 21 (http://www.mirbase.org/) [22, 23], using Bowtie (v 1.1.0) [24]. Only the reads exactly matched to mature -5p/-3p of the miRNA precursors without mismatch and allowing extending or shortening by 2bp, were considered for known miRNAs. RNA editing or SNPs for these miRNAs was then detected through alignment to the goat miRNA precursors with one mismatch. Since the sequence of mature miRNA might not be identical in different animal species, the reads retained from the last two steps were aligned to mature -5p/-3p of the recorded miRNA precursors of other animal species in the miRBase 21 with two mismatches and allowing extending or shortening by 2bp to identify goat miRNA homologs. However, it should be noted that these miRNAs likely were a mixture of the miRNAs homologs and the known RNAs with modifications of the 3´ nucleotide additions or RNA editing. The raw count for known miRNAs and miRNA homologs were summarized and then normalized using reads per million mapped reads (RPM). In order to improve accuracy, only those miRNAs or homologs with an overall expression level greater than one RPM in eight libraries were finally considered as the true known miRNAs or miRNA homologs. Mullokandov et al. [25] suggested that a miRNA should be at a minimum of 100 RPM for activity, and that 80% of miRNAs above 1000 RPM were functional. We thus divided these miRNAs into a low expression group (< 100 RPM), a moderate expression group (≥ 100 to 1,000 RPM) and a high expression group (≥ 1,000 RPM), based on overall average RPM values.

Total clean reads were also mapped to the goat reference genome (CHIR_1.0) [26] (ftp://ftp.ncbi.nlm.nih.gov/genomes/) using the Bowtie aligner. The mapped reads were further annotated to the repeats and exon regions based on the genome annotation, and subsequently filtered against all the annotations mentioned above. The unannotated reads that could not be matched to any public databases or the reference genome were used to predict potential novel miRNA using Mirdeep2 (v2.0.0.7) [27]. Specifically, the miRDeep2.pl script in the miRDeep2 software was applied to identify novel miRNA using default options (e.g. minimal free energy < -18 kcal/mol). This analysis would result in a scored list of novel miRNAs with log-odds score, which could help us find genuine novel miRNAs. To achieve high accuracy, only those miRNAs with a miRDeep2 score higher than or equal to five, and an estimated RNA-fold P-value of less than 0.05, were retained as true novel miRNAs. The MIREAP software (http://sourceforge.net/projects/mireap/) was also employed to predict novel miRNAs with these default parameters. Finally, the intersection of the predicted miRNAs from miRDeep2 and MIREAP were considered to be the genuine novel miRNAs.

### Differential expression analysis of the identified miRNAs

We applied upper quartile (UQ) scaling in the edgeR package [28] to the raw read counts to more accurately compare the expression profiles of identified miRNAs across different libraries, according to the method proposed by Tam et al. [29]. The differentially expressed (DE) miRNAs were identified through pairwise comparisons between every two stages. The significantly DE miRNAs were determined according to the following criteria: (1) the change in expression levels was above or equal to two-fold (|log2 (Fold change)| ≥ 1) and (2) the corrected *P-value* (FDR) ≤ 0.05.

In order to explore the co-expression profiles of the DE miRNAs, STEM software (v 1.1) [30] was used to cluster and visualize expression patterns of DE known miRNAs. The maximum unit change in model profiles between time points was adjusted to 1 and the maximum number of model profiles to 30. Expression profiles of miRNAs were clustered based on their log_2_ (RPM values) and their correlation coefficients. The statistical significance of the number of miRNAs to each profile versus the expected number was computed using algorithm proposed by Ernst et al [30].

### Identification and Function annotation of miRNA targets

All putative 3' untranslated regions (UTR) of the goat mRNAs extracted from the reference genome (CHIR_1.0), were independently utilized for the prediction of miRNA target genes independently via miRanda [31], TargetScan 7.0 [32], and RNAhybrid v2.1.2 [33]. Intersections of the results from the three programs comprised the final predicted gene targets. However, we focused on the function significance of the highly and moderately expressed DE miRNAs (overall average expression > 100 RPM) among every pairwise comparisons, based on the relationships between functionality and expression abundance [25]. We also obtained the transcriptome data in *longissimus dorsi* of the same sampled animals (unpublished data). We finally selected the putative target genes that showed opposite expression changes to the DE miRNAs. Gene enrichment in the Gene Ontology (GO) biological processes and KEGG pathways databases were performed using the DAVID v6.7 [34] (http://david.abcc.ncifcrf.gov/). Human homologous gene symbols of the putative gene targets were used for downstream bioinformatics analysis, because there was no goat genome information available in DAVID.

### Quantitative RT-PCR validation of DE miRNAs

Quantitative real-time PCR was used to verify the reliability of the expression profiles of DE miRNAs. Nine miRNAs were finally selected based on the differential expression patterns of these miRNAs across the four time points and their TPM values. Total RNA was isolated with the Trizol reagent (Invitrogen, CA, USA) according to the manufacturer’s instructions.The first strand cDNA was synthesized using the Mir-X miRNA First-Strand Synthesis Kit and SYBR^®^ Premix Ex Taq^™^ Tli RNase H Plus (Takara) was used for qPCR. The qPCR was carried out with a total volume of 10 μL containing 5 μL 2 × SYBR Premix Ex Taq II, 0.4 μL primers (10 μM), and 0.8 μL diluted cDNA. The PCR conditions were as following: denaturation at 95°C for 30 s, followed by 40 cycles of 94°C for 15 s, annealing temperature for 30 s, and 72°C for 30 s. A melting program ranging from 55 to 95°C with a heating rate of 0.5°C per10 s was carried out to create the melting curves. U6 snRNA was selected to normalize the expression levels and relative gene expressions were calculated by the 2-ΔΔCt method. The qPCR primers for nine selected miRNAs are listed in S10 Table. The Pearson correlation coefficients (r) for relative expression abundance of each selected miRNAs were caculated to assess the consistency of both approaches.

## Results

### Deep sequencing of small RNAs in goat *longissimus dorsi*

**Fig 1:**
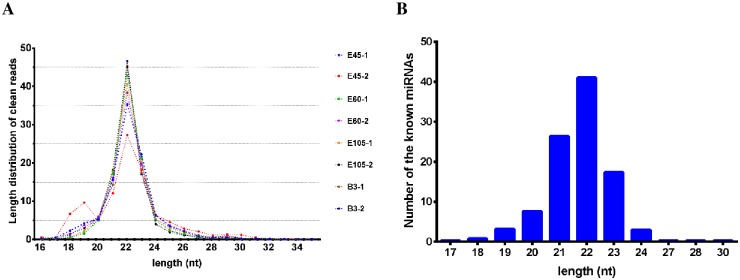
Basic analysis of the sequencing data. (A) Sequence length distribution of clean reads; (B) Sequence length distribution of known miRNAs.

To identify miRNAs that act as regulators of goat skeletal muscle development, a total of 110,614,744 raw reads with an average of about 13.8 million reads per sample were first generated from eight miRNA libraries at four time points (S1 Table). After removing low-quality sequences, sequences shorter than 16 nt, and contaminants formed by adapter–adapter ligation, a total of 94,692,697 clean reads were retained for further analysis (S1 Table). The sequence lengths of the clean reads in the eight small RNA libraries were almost distributed as 18–30 nt, and the largest percentage (27.29%–46.57%) was 22 nt, followed by 23 nt and 21 nt, as shown in Fig 1A, indicating good sequencing quality. To detect known goat miRNAs, 33.79–63.58% of the total clean reads of each library were aligned to the goat miRNA precursors in miRBase 21 (S1 Table). In addition, about 68% (61.61–74.20%) of the clean reads per library were mapped to the goat reference genome (S1 Table). The mapped reads were further aligned to the repeats and exon regions to filter out the reads encoded by these sequences. Ultimately, the remaining unannotated reads were used to identify novel miRNAs.

### Several known miRNAs were overrepresented in goat *longissimus dorsi*

We detected a total of 410 known goat miRNAs with a wide range of raw counts, from six reads for the least abundant to 5,666,981 reads for the most abundant in the eight libraries, as shown in S2 Table. The most abundant size class of these miRNAs was 22 nt (40.98%), followed by 21 nt (26.34%) (Fig 1B). Considering RPM as a unit, the overall average expression level of the known miRNAs varied from 0.13 to 74,822 RPM with a median of 80 RPM and a mean of 1,723 RPM. The number of the lowly (< 100 RPM) and moderately (≥ 100 RPM to 1,000 RPM) expressed miRNAs accounted for 52.68% and 28.29% of the known RNAs, respectively (S2 Table). In contrast, the proportion of the known miRNAs with high abundance was just 19.02%, among which 19 miRNAs (4.63%) expressed over 10,000 RPM. Furthermore, the number of low abundant miRNAs increased throughout the developmental stages. The expression of highly expressed miRNAs comprised up to 93.29% of all known miRNAs. In particular, the top 19 expressed miRNAs accounted for 63.44% of the total expressed miRNAs (Table 1), indicating that a few miRNAs were over-represented in our small RNA libraries. The most abundant miRNA was chi-miR-1, which was highly expressed throughout the four sampled stages and detected 598,572.64 RPM (5,387,272 raw counts) in eight libraries, encompassing 10.59% of all known miRNAs. This result was consistent with the established roles of miR-1 during skeletal muscle development [3]. MiR-206, which regulates myogenic differentiation by repressing PAX7 [35], was the second most abundant miRNA, followed by chi-miR-136-5p and chi-miR-125b-5p. Three members (chi-let-7f-5p, chi-let-7a-5p, and chi-let-7i-5p) of the let-7 family, which is thought to be ubiquitously expressed in a variety of animal tissues, were also highly expressed across the four sampled stages. In addition, the average expression of chi-miR-133a-3p was still as high as 10,974.55 RPM.

**Table 1:** Most abundant 19 known miRNAs across all four stages (RPM>10000).

miRNA Name	E45 RPM	E60 RPM	E105 RPM	B3 RPM	Overall average RPM	Percent of total RPM (%)
chi-miR-1	6295.55	46071.45	57320.36	189598.96	74821.58	10.59
chi-miR-206	14431.74	42889.82	43362.45	45839.08	36630.77	5.18
chi-miR-136-5p	24987.69	39717.23	63624.06	17124.32	36363.32	5.15
chi-miR-125b-5p	75912.97	27792.61	22774.97	18196.78	36169.33	5.12
chi-miR-199a-5p	60496.56	39203.87	22301.77	10368.77	33092.74	4.68
chi-miR-127-3p	19427.97	32673.96	50650.07	14755.67	29376.92	4.16
chi-miR-378-3p	13468.09	18796.43	17111.45	35972.47	21337.11	3.02
chi-miR-199a-3p	32368.72	20436.40	14383.53	8331.90	18880.14	2.67
chi-let-7f-5p	8267.54	25391.62	19610.74	21285.13	18638.76	2.64
chi-miR-381	1163.04	15798.25	39639.43	16014.21	18153.73	2.57
chi-let-7a-5p	12391.40	21288.32	20529.56	16818.11	17756.85	2.51
chi-miR-26a-5p	11827.52	15451.15	14309.29	23221.40	16202.34	2.29
chi-miR-424-5p	9682.27	16680.34	24418.99	11053.80	15458.85	2.19
chi-miR-542-3p	17859.67	18067.31	15563.14	4943.97	14108.52	2.00
chi-miR-21-5p	9380.94	17679.31	17354.65	9719.30	13533.55	1.92
chi-let-7i-5p	12745.99	18266.53	12772.41	5858.31	12410.81	1.76
chi-miR-101-3p	10321.09	11493.82	10860.86	16368.79	12261.14	1.74
chi-miR-99a-5p	16573.32	13034.98	10945.55	7581.35	12033.80	1.70
chi-miR-133a-3p	3148.03	6608.13	8574.40	25567.65	10974.55	1.55

### A large miRNA cluster on chromosome 21 expressed in goat *longissimus dorsi*

**Fig 2:**
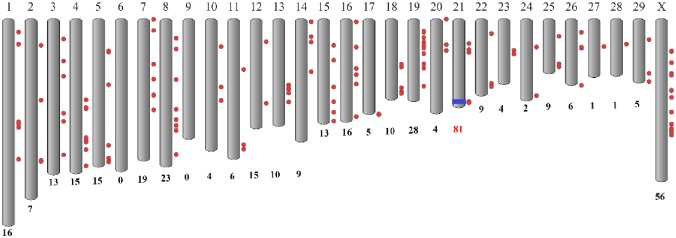
Genomic distribution of the known miRNAs across goat chromosomes. The numbers at the bottom of the chromosome schematic bars represent amounts of miRNAs detected on each chromosome. Red circles at the right of the chromosomes represent the known miRNAs located on the chromosomes. The blue bar on chromosome 21 represents the miRNA cluster at 62.0 Mb—63.0 Mb region. It is noteworthy that many miRNAs on the same chromosome (i.e. the miRNA cluster on chromosome 21) are compactly arrayed and may not be distinguishable in this figure due to their close proximity.

**Fig 3:**
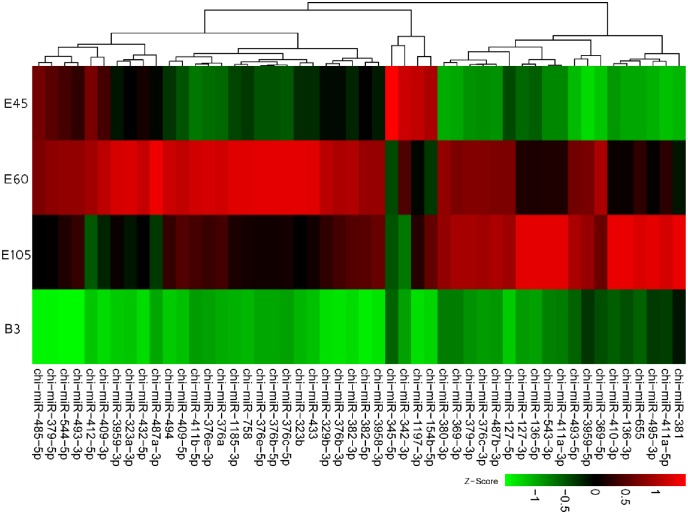
The expression heat-map of 50 miRNAs (> 100 RPM) locating at 62.0 Mb—63.0 Mb region of chromosome 21 across all four stages based on the RPM values.

We also investigated the genomic locations (CHIR_1.0) for the 410 known miRNAs identified above (S2 Table). Most (402) of these miRNAs were distributed throughout all the goat autosomes and the X chromosome, with the exception of chromosome 6 and 9 where no known miRNAs were located (Fig 2). However, eight miRNAs did not match any genomic sequences, which might be caused by an incomplete assembly model of the goat genome (S2 Table). Remarkably, chromosome 21 encoded the highest number (81) of known miRNAs, accounting for 19.75% of these miRNAs. After culling the lowly expressed miRNAs, 50 miRNAs with overall average RPM larger than 100 were found to aggregate at the 62.0 Mb—63.0 Mb (~ 1 Mb) short region of chromosome 21 (S2 Table and Fig 2). According to the goat reference genome, this miRNA cluster was located at the imprinted *DLK1–DIO3* locus. Fig 3 displayed that these 50 miRNAs were classified into two sub-clusters at the highest hierarchical level based on their RPM values across all four stages, which substantially matched their chromosomal locations. The first sub-cluster mainly included chi-miR-127-5p/3p and chi-miR-381, and the second sub-cluster comprised chi-miR-379-5p and chi-miR-544. Interestingly, the average expressions of chi-miR-379-5p was as high as about 9,400 RPM. Chromosome X encoded the second highest number (56) of known miRNAs which were scattered in different short regions. Some of these miRNAs showed very high expression levels with larger than 10,000 RPM (chi-miR-424-5p and chi-miR-542-3p). In contrast, only one known miRNA was present in both chromosome 27 and 28. The longest (1) and shortest (25) chromosome, encoded 16 and 9 miRNAs, respectively.

### miRNA homologs ubiquitously expressed in *longissimus dorsi*

Based on the alignment against precursor miRNAs of other animal species in miRBase 21 with two mismatches, we detected a total of 752 miRNA homologs (S3 Table). Although most (86.70%) of these miRNA homologs were expressed at a low level, 24 homologs were on average highly abundant (≥ 1,000 RPM). In particular, the overall average expression of miR-181-5p, which is the same as mmu-miR-181a-5p in sequence, was up to 27618.52 RPM across all four stages, a results that is consistent with the functions of miR-181a in myoblast differentiation [36]. According to the alignment, eight highly expressed miRNA homologs (e.g. miR-181-5p, miR-152-3p, and miR-299a-5p) had identical sequence as the mature miRNAs in mouse or human recorded in the miRBase 21 (S3 Table). We, thus, annotated these miRNAs as goat miRNAs. However, numerous miRNA homologs were only different in the 3´ end position with the known goat sequences, which was possibly due to base modification. For example, the sequences of miR-127-3p and chi- miR-127-3p were only different at the 3´ end with CTT bases, and miR-125-5p could be produced from chi-miR-125b-5p through the addition at the 3´ of three nucleotides (GAA).

### Novel miRNAs lowly expressed during skeletal muscle development

By selecting the intersection of novel miRNAs predicted by the mirDeep2 (149 miRNAs) and MIREAP (490 miRNAs) software, a total of 88 putative novel mature miRNAs encoded from 90 precursor sequences were identified across the eight libraries. These were named chi-miR-new-N (N = 1 to 88) (S4 Table). These novel miRNAs ranged in size from 20 nt to 24 nt, among which 22 nt sequences accounted for the largest proportion (54.55%), followed by 21 nt (21.59%), and 23 nt (15.91%). The size of all these novel miRNAs was consistent with the typical length distribution of mature miRNAs. In addition, these miRNAs were distributed across 24 chromosomes and one scaffold, with the largest number (8) on chromosome 13. Considering total raw counts in eight libraries, the novel miRNAs were expressed at a relatively low level: only three mature miRNAs were beyond 1000 raw counts. Intriguingly, chi-miR-new-1 processed from two different precursors on chromosome 17 was totally expressed at about 10000 raw counts, which was reserved for further analysis.

### Differential expression profiles of miRNAs across four developmental stages

**Fig 4:**
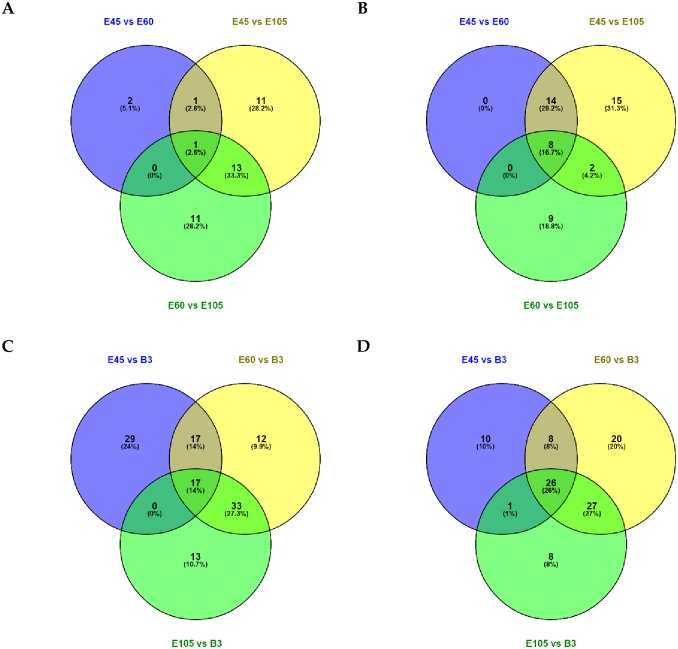
Number of commonly and uniquely DE miRNAs between the different stages of pairwise comparisons. Number of up-regulated (A) and down-regulated (B) miRNAs among prenatal stages (E45 vs E60, E45 vs E105 and E60 vs E105). Number of down-regulated (C) and up- regulated (D) miRNAs between the neonatal stage and prenatal stages (E45 vs B3, E60 vs B3, and E105 and B3). Differentially expressed miRNAs were detected using the cutoff values of |log2 (Fold change)| > 1 and FDR < 0.05. Here the fold changes were calculated from the pseudo-counts based on the UQ normalizations which was different from the fold changes based on RPM values of the miRNAs.

The pairwise Pearson correlation coefficients between the libraries were calculated using the RPM values of the known miRNAs. The lowest correlation (0.2116) was observed between the E45-2 and B3-2 libraries, whereas the B3-1 library had the highest correlation with library B3-2 (S5 Table). Although many miRNA homologs had significant differential expression, we focused on the known goat miRNAs. A total of 221 DE known miRNAs (FDR < 0.05) were detected across four developmental stages (Fig 4 and S6 Table), according to UQ normalization. In particular, 40 of the highly expressed known miRNAs (average RPM > 1000) were significantly DE among all the pairwise comparisons, which included miR-1, miR-133a-3p and miR-499-5p (S6 Table). It was clear that the number of DE miRNAs among the three prenatal stages was much lower, compared to that between any given prenatal stage and the neonatal stage. For instance, the highest number of DE miRNAs (160) was found between E60 and B3, including 81 up-regulated and 79 down-regulated miRNAs. In contrast, the comparison between the E45 and E60 gestation stages only identified 4 up-regulated and 22 down-regulated miRNAs, which was the least among all comparisons. Venn diagrams (Fig 4A and 4B) showed that only one down-regulated and eight up-regulated miRNAs were common in comparisons of all prenatal stages. However, 17 down-regulated miRNAs were common between three prenatal stages and the neonatal stage, including chi-miR-409-3p, chi-miR-376b-3p, and chi-miR-135a. Twenty-six commonly up-regulated miRNAs were present in the comparisons of three prenatal stages and the neonatal stage, including chi-miR-1, chi-miR-378-3p and chi-miR-26a-5p (Fig 4D). In addition, 50% of the up-regulated miRNAs for E45 vs. E105 were part of the miRNA cluster located on chromosome 21. Among all DE known miRNAs, chi-miR-183 sufferedthe largest down-regulation (-8.41) in comparison to E45 vs E105, while chi-miR-1 benefited from the highest up-regulation (6.08) between E45 and B3. Interestingly, 21 of the 22 down-regulated miRNAs showed more than three-fold increase from E45 to E60, including miR-124a, miR-9-3p, and miR-381 (S6 Table). Although many DE miRNAs were identified in the comparison of E60 vs E105 (44) and E105 vs B3 (125), the majority showed moderate fold changes (< 2-logFC) of gene expression.

**Fig 5:**
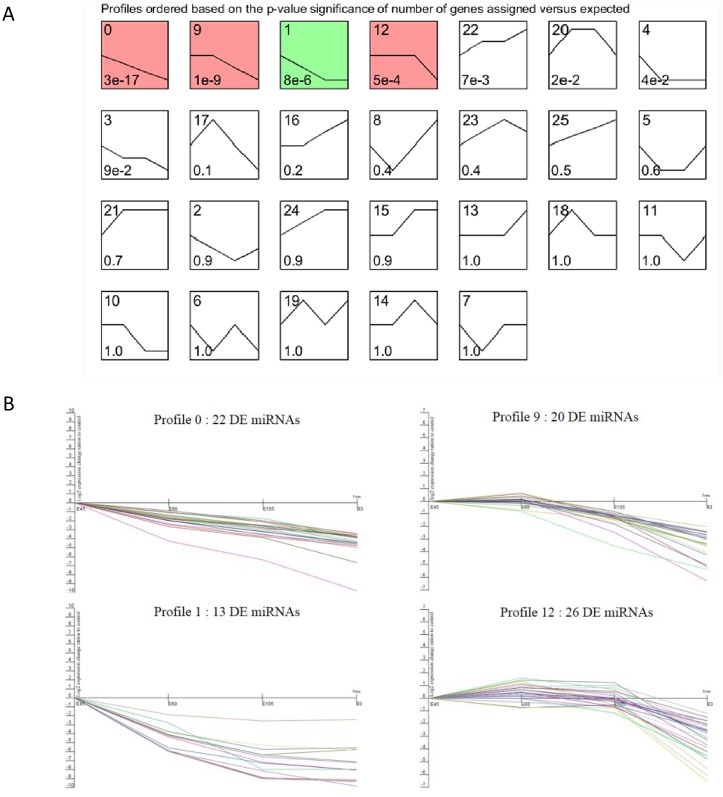
STEM analysis of the DE known miRNAs expression profiles. (A) Each box corresponds to a type expression profile and only colored profiles are statistically significant. The upper-left and bottom-left numbers in each box indicate the order of profiles and P-values, respectively. (B) Four significant clusters of miRNA profiles across all four stages.

The co-expression clustering analysis for the DE known miRNAs revealed that a total of 81 DE known miRNAs were clustered significantly into four types of expression profiles (Fig 5 and S7 Table). For example, 22 continuously down-regulated miRNAs (e.g. miR-196a/b) across all sampled stages were significantly enriched into Profile 0 (Fig 5), whereas the miRNAs showing gradually increasing expression (miR-1/miR-133a-3p/miR-133a-5p) did not cluster significantly. In particular, the expressions of 26 DE known miRNAs in Profile 12 were stable at prenatal stages and then went down on day three after birth, such as chi-miR-127-5p, chi-miR-409-3p, and chi-miR-432-5p. Furthermore, 20 DE miRNAs in Profile 9 including chi-miR-19a, chi-miR-3431-5p, and chi-miR-412-5p, expressed relatively higher at E45 and 60 stages followed by a decline at later sampled stages.

### Target genes of the DE miRNAs reflected the temporal change of goat skeletal muscle development

**Fig 6:**
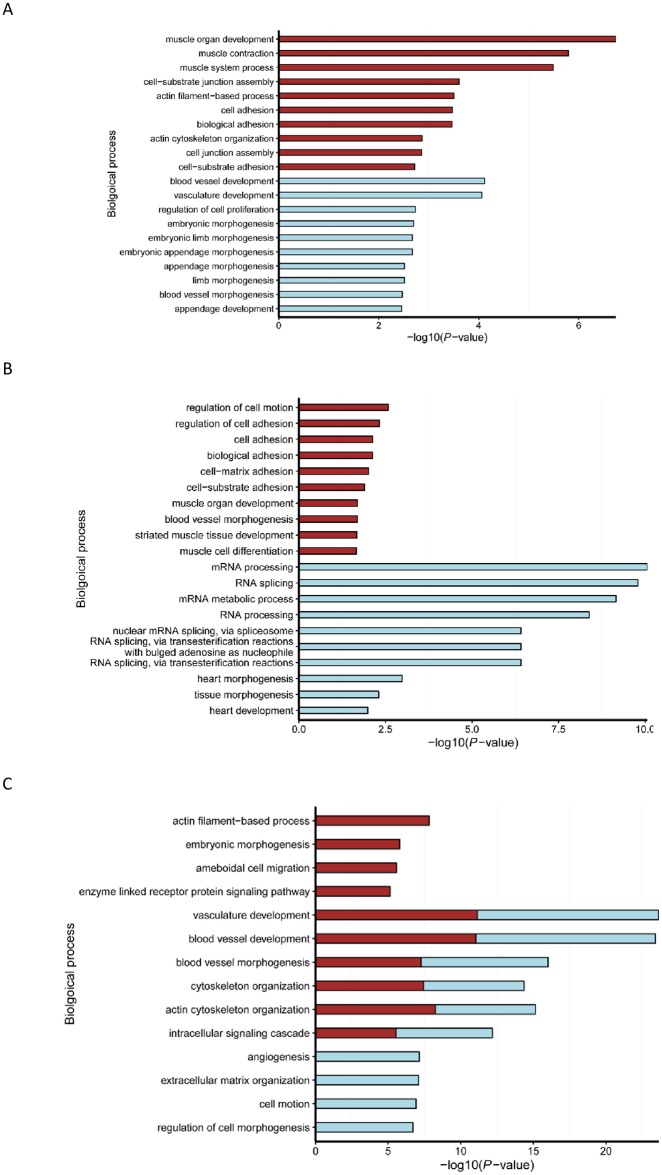
Top ten GO biological processes enriched with DE miRNAs (overall average expression > 100 RPM) in the three adjacent comparisons. A: E45 vs. E60, B: E45 vs. E105, C: E45 vs. B3. The X-axis represents the score for the likelihood [−log (*P*-value < 0.05)] that genes belong to a specific functional group. The Brown bar represents enriched GO terms associated with down-regulated miRNAs, while the light blue bar represents enriched GO terms associated with up-regulated miRNAs.

The GO enrichment analysis of genes targeted by the DE miRNAs for all the pairwise comparisons generated a large number of significant (*P* < 0.05) annotation results (S8 Table). However, in order to examine their roles in temporal changes of goat skeletal muscle development, we mainly provided detailed descriptions of the results for the DE miRNAs in the three adjacent comparisons. As shown in Fig 6A, the top ten processes (*P* < 0.05) significantly related to genes targeted by the down-regulated miRNAs in the E45 vs. E60 comparison. These mainly included muscle organ development, muscle contraction, cell-substrate junction assembly, and cell adhesion. In the E60 vs. E105 comparison, the most significantly enriched processes for the down-regulated miRNAs were also associated with biological adhesion and muscle development. These were the following: regulation of cell adhesion, cell-matrix adhesion, cell-substrate adhesion and striated muscle tissue development (Fig 6B). Interestingly, genes targeted by up-regulated miRNAs in this comparison were most significantly associated with mRNA transcriptions (Fig 6B), including mRNA processing, RNA splicing, mRNA metabolic process, RNA processing, and nuclear mRNA splicing via spliceosome. In the E105 vs. B3 comparison, the top ten processes for the down-regulated and up-regulated miRNAs were mainly associated with vasculature development, blood vessel development, and bone development (Fig 6C).

All significantly enriched KEGG pathways (*P* < 0.05) of DE miRNAs are also provided in S9 Table. Comparing E45 vs. E60 and E60 vs. E105, only four and two pathways were significantly associated with the genes targeted by DE miRNAs, respectively. Specifically, the enriched pathways for E45 vs. E60 mainly included ECM-receptor interaction and focal adhesion, which are well-known for their functions in cell fusion and tissue junctions. In the comparison of E60 vs. E105, only the spliceosome pathway were significantly enriched for the up-regulated miRNAs, which is in accordance with GO annotations. In contrast, large numbers of pathways were significantly related to the DE miRNAs for E105 *vs*. B3. The overlapping pathways for the up-regulated and down- regulated miRNAs contained ECM-receptor interaction, DNA replication, TGF-beta signaling, cell cycle, adherens junction, and p53 signaling. By examining the annotation results for E45 *vs*. B3 and E60 *vs*. B3, we found the enriched pathways to be similar for these three comparisons, possibly indicating dramatic changes of skeletal muscle development between prenatal stages and a neonatal stage. It was also notable that fatty acid pathways were significantly enriched for E45 vs. B3 and E60 vs. B3, implying more activity of the fatty acid metabolism during late gestation and postnatal stages.

### Quantitative RT-PCR validation of DE miRNAs

**Fig 7:**
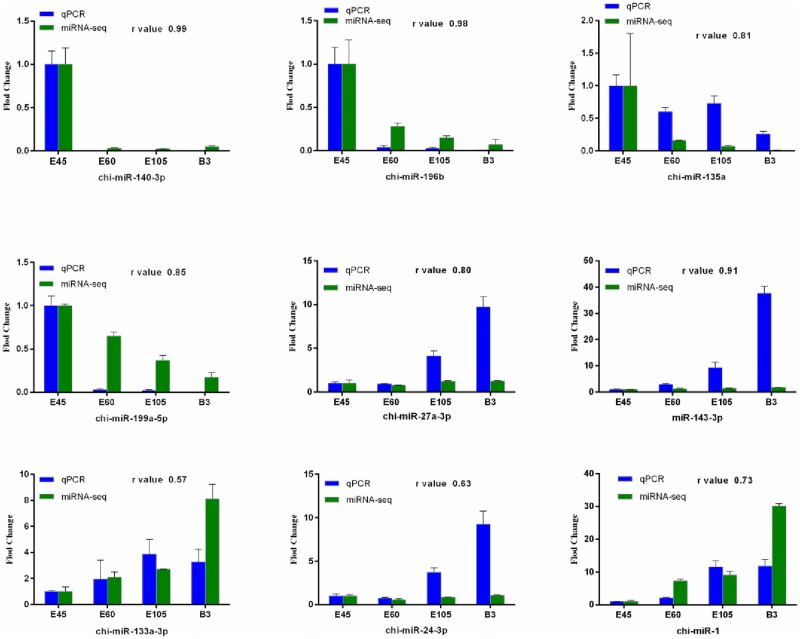
qPCR validation of miRNA expression in skeletal muscle samples. The r value represents the Pearson Correlation Coefficient between two methods. The abundance of the miRNAs is normalized to abundance of U6 snRNA.

In order to verify the high-throughput sequencing data, real-time quantitative PCR (qPCR) was used to validate the expressed levels of nine selected DE miRNAs. These were chi-miR-1, chi-miR-196b, chi-miR-140-3p, chi-miR-199a-5p, chi-miR-27a-3p, chi-miR-143-3p, chi-miR-135a, chi-miR-133a-3p, and chi-miR-24-3p. The relative expression changes of qPCR results showed a highly positive correlation (r = 0.57–0.99) to the comparatively small RNA sequencing data (Fig 7), suggesting reliability of the small RNA-sequencing approach.

## Discussion

Skeletal muscle development is a complex biological process controlled by numerous protein-coding genes and non-coding RNAs, including miRNAs [10, 11]. Although advances have been made regarding the actions of miRNAs during goat [21] and sheep [15, 37] myogenesis, the expression landscape of miRNAs during goat skeletal muscle development remains poorly understood. Accordingly, in order to provide new sights into the functions of miRNAs during goat skeletal development, we systematically identified the miRNAs that regulate goat skeletal myogenesis, and examined their temporal expression profiles in *longissimus dorsi* muscle from an early prenatal stage to a neonatal stage.

The prenatal stage is crucial for skeletal muscle development in animals, mainly because almost all muscle fibers are formed during this period and there is no increase in the number of fibers after birth [9]. Previous studies in livestock have demonstrated that many miRNAs are highly expressed in prenatal stages and thus are thought to play important roles in skeletal myogenesis [12, 14, 16, 21]. In this work, a total of 410 known goat miRNAs and 752 goat miRNA homologs were identified across the four developmental stages studied. Some of the expressed miRNAs identified here have already been reported in different species [3, 14, 16, 21, 38], indicating similar molecular mechanisms underlying skeletal muscle development in animals. For instance, eleven and nine of the most abundant miRNAs (over 10000 RPM), including the myomiRs, were among the top 20 abundantly expressed miRNAs detected during skeletal muscle development in porcine [14] and Xuhuai goat [21], respectively. In addition to myomiRs, previous studies have revealed the roles of other abundantly expressed miRNAs in skeletal myogenesis. For example, miR-378-3p could increase the expression of *MyoD* by repressing *MyoR* in myoblast cells [39]; miR-424 promoted C2C12 myoblast differentiation via direct targeting of the 3´UTR of *Cdc25A* [40]. Ge et al. [41] have reported that miR-125b-5p negatively modulated muscle regeneration in mice, while *IGF-2* is a direct target of miR-125b-5p. Furthermore, chi-miR-181-5p was highly expressed throughout the sampled development stages, which is consistent with increasedabundance of miR-181a in skeletal muscle tissues of young animals [42]. However, the functions of other miRNAs that are abundant during skeletal muscle development, such as chi-miR-412-5p and chi-miR-542-3p, remained largely unknown.

Although most animal miRNA genes are widely scattered throughout the genome, increasing evidence indicates that clustered miRNAs are expressed from the same primary transcripts with similar pattern [43, 44]. Here, we found that chromosome 21 encoded the highest number (81) of known miRNAs in *longissimus dorsi*. Surprisingly, 50 miRNAs (> 100 RPM) located in a short region at about 62.0 Mb—63.0 Mb were divided into two sub-clusters, which mainly included chi-miR-136-5p/3p, chi-miR-127-5p/3p and chi-miR-379-5p. According to the genome information from Ensemble, these miRNA homologs were clustered at 64 Mb—65 Mb on sheep chromosome 18 (Oar_v3.1), 67 Mb—68 Mb on cattle chromosome 21 (UMD3.1), and 100 Mb—101 Mb on human chromosome 14q32 (GRCh38.p5). This region resides at the imprinted *DLK1–DIO3* domain in the mammal genome [44]. Previous studies have demonstrated that the imprinted *DLK1–DIO3* locus is conserved in animals, and is important in epigenetic and gene regulation during development [45]. This miRNA cluster was also detected in *longissimus dorsi* of the *Callipyge* sheep [37], suggesting an inhibitory effect of the miRNAs on the expression of *DLK1*. Moreover, this miRNA cluster on the mouse distal chromosome 12 was down-regulated in mouse skeletal muscles due to aging [46]. Recently, deletion of the maternally expressed miR-379/miR-544 cluster that is highly expressed in embryonic and neonatal stages, led to fast-twitch muscle hypertrophy via regulation of *DLK1* expression in mice [47]. Therefore, this miRNA cluster might play fundamental roles during muscle development in a yet unknown way.

Deep sequencing has become the main approach to detect novel miRNAs and isomiRs [48]. As two mismatches were allowed, the identified miRNA homologs in our study also included the variants of the known goat miRNA that result from modifications of the 3´nucelotide additions, which occur ubiquitously in animals [48, 49]. For example, miR-125-5p could be generated from chi-miR-125b-5p through addition of three 3´ nucleotides. In addition, some miRNA homologs (e.g. miR-127-3p, miR-125-3p, and miR-143-3p) show a slight difference to the mature sequence of the known goat miRNAs and also had abundant expressions (> 1000 RPM). However, additional follow-up work is needed to ascertain the detailed patterns of miRNA modifications during goat skeletal muscle development. The combination of two softwares with stringent parameters helped us to accurately discover 88 putative novel miRNAs. Accordingly, our miRNA homologs and novel miRNAs would greatly increase the public repertoire of goat miRNAs.

In this study, we mainly focused on the dynamic expression profiles of the known goat miRNAs, while some miRNA homologs showed significantly differential expressions. Based on the UQ normalization, a total of 221 DE known miRNAs were identified across the four developmental stages [28]. Remarkably, many known miRNAs showed significant difference in expression between the three prenatal stages and the neonatal stage. For instance, 125 miRNAs showed differential expression between E105 and B3, whereas there were only 26 DE miRNAs in the E45 vs E60 comparison. In addition, the expression of several miRNAs (overall average miRNAs > 100 RPM) was relatively stable during the prenatal stages but then decreased on day three after birth. These included chi-miR-127-5p, chi-miR-409-3p, and chi-miR-432-5p. These results collectively reflect that the functions of miRNAs changed fundamentally from prenatal stages to neonatal stages. Wang et al. [21] recently showed that chi-miR-432-5p was expressed much more abundantly in a 90-day-old fetus compared to a nine-month-old goat. Our results indicated the persistent actions of miR-432-5p in goat embryonic myogenesis. In addition, miR-196a/b was continuously expressed until fetal day 45, when it started to decrease. This is consistent with their roles of modulating body axial patterning during early embryonic stages [50]. The continuously up-regulated expression patterns of miR-1, miR-206, miR-133 and miR-378 were similar to those in pig [14], which is consistent with their critical roles during muscle development. Although McDaneld et al. [12] reported that miR-381 was specifically expressed on day one after birth, chi-miR-381 in this study was abundant throughout the prenatal stages with the expression peaking after the prenatal stage (E105). This indicates that miR-381 also plays an important role in fetal muscle growth.

In this study, the functional enrichment analysis revealed some DE miRNAs to be involved in the temporal changes of goat skeletal muscle development. For instance, the muscle development and biological adhesion related processes were regulated by down-regulated miRNAs. This probably reflects that differentiation and fusion of the myoblast were very active between day 45 and day 60 of gestation. This result is consistent with the generation of muscle cells in fetal sheep [51]. In contrast, the RNA transcription related processes were enriched for up-regulated miRNAs, which might be an indication that protein synthesis in skeletal muscle was temporarily reduced. This finding was supported by the results in the matured skeletal muscle at approximately d 105 of gestation in sheep [52], considering that the gestation period of goat and sheep is on average 150 days for each pregnancy. The adipogenesis of intramuscular fat occurs during late gestation in mammals, such as pig and sheep. Here, the adipogenesis related GO processes were enriched for the target genes between E105 and B3, implying that the miRNAs were also involved in adipogenesis of goat intramuscular fat in prenatal stages. The miRNAs that corresponded to the target genes involved in the abovementioned biological actions would be our candidate miRNAs for further studies on goat skeletal development.

## Conclusions

In the present study, we systematically identified the miRNAs involved in goat skeletal muscle development and investigated their temporal expression profiles during muscle development across three goat prenatal stages and one neonatal stage, using high-throughput sequencing technology. A total of 410 known goat miRNAs, 752 miRNA homologs and 88 novel miRNAs were identified in *longissimus dorsi* with a wide range of expression levels. In particular, 19 known goat miRNAs as well as miR-181-5p were highly expressed with an average abundance larger than 10,000 RPM. Furthermore, 50 of the known miRNAs showing more than 100 RPM were located at the *DLK1–DIO3* region of goat chromosome 21. Our results suggest that a high number of miRNAs are involved in goat skeletal muscle development. Moreover, 221 known miRNAs were significantly DE across the sampled stages, particularly between the prenatal stages and the neonatal stage. The functional enrichment analysis suggests the biological pathways regulated by the identified miRNAs to be directly linked to the temporal changes of goat skeletal muscle development.

## Supporting Information

S1 Table Annotations of sequenced small RNAs. (XLSX)

S2 Table Summary of reads distribution and expression profiles of the known goat miRNAs. (XLSX)

S3 Table Summary of reads distribution and expression profiles of miRNA homologs. (XLSX)

S4 Table Summary of predicted novel miRNAs. (XLSX)

S5 Table Pearson correlations among eight libraries based on the known miRNAs. (XLSX)

S6 Table Differentially expressed known goat miRNAs across four stages. (XLSX)

S7 Table Four significant expression profiles for DE known miRNAs. (XLSX)

S8 Table Significantly enriched GO pathways for the targets of DE known miRNAs. (XLSX)

S9 Table Significantly enriched KEGG pathways for the targets of DE known miRNAs. (XLSX)

S10 Table The miRNA primers of used for qPCR validation. (XLSX)
